# Pretargeted adjuvant radioimmunotherapy with Yttrium-90-biotin in malignant glioma patients: A pilot study

**DOI:** 10.1038/sj.bjc.6600047

**Published:** 2002-01-21

**Authors:** C Grana, M Chinol, C Robertson, C Mazzetta, M Bartolomei, C De Cicco, M Fiorenza, M Gatti, P Caliceti, G Paganelli

**Affiliations:** Division of Nuclear Medicine, European Institute of Oncology, via Ripamonti 435, I-20141, Milano, Italy; Division of Epidemiology and Biostatistics, European Institute of Oncology, via Ripamonti 435, I-20141, Milano, Italy; Department of Pharmaceutical Science, University of Padova, Italy

**Keywords:** glioma, radioimmunotherapy, avidin–biotin

## Abstract

In a previous study we applied a three-step avidin–biotin pretargeting approach to target ^90^Y-biotin to the tumour in patients with recurrent high grade glioma. The encouraging results obtained in this phase I–II study prompted us to apply the same approach in an adjuvant setting, to evaluate (i) time to relapse and (ii) overall survival. We enrolled 37 high grade glioma patients, 17 with grade III glioma and 20 with glioblastoma, in a controlled open non-randomized study. All patients received surgery and radiotherapy and were disease-free by neuroradiological examinations. Nineteen patients (treated) received adjuvant treatment with radioimmunotherapy. In the treated glioblastoma patients, median disease-free interval was 28 months (range=9–59); median survival was 33.5 months and one patient is still without evidence of disease. All 12 control glioblastoma patients died after a median survival from diagnosis of 8 months. In the treated grade III glioma patients median disease-free interval was 56 months (range=15–60) and survival cannot be calculated as only two, within this group, died. Three-step radioimmunotherapy promises to have an important role as adjuvant treatment in high grade gliomas, particularly in glioblastoma where it impedes progression, prolonging time to relapse and overall survival. A further randomized trial is justified.

*British Journal of Cancer* (2002) **86**, 207–212. DOI: 10.1038/sj/bjc/6600047 www.bjcancer.com

© 2002 The Cancer Research Campaign

## 

Despite years of intensive research, the prognosis for high grade gliomas (grade III glioma and glioblastoma (GBM)) remains conspicuously poor. Median survival for adults with glioblastoma is 8–12 months after diagnosis (Walker *et al*, 1985). The outlook is somewhat better for grade III glioma with a 38–50% 2-year survival rate. However, primary high grade brain tumours are highly resistant to currently available therapies. Occasionally responses to surgery, radiotherapy or single-multiple chemotherapy are observed in the setting of recurrent tumours, but these responses are usually of short duration, resulting in a brief survival time. Thus, new treatments, such as gene therapy (Culver *et al*, 1992; Roth and Cristiano, 1997) and the use of monoclonal antibodies (MoAbs) in association with cytotoxic agents are being investigated (Zalutsky *et al*, 1989).

Intralesional radioimmunotherapy (RIT) for high grade gliomas using anti-tenascin monoclonal antibodies labelled with iodine-131 has been used with encouraging results and without major side effects (Riva *et al*, 1994). However, the limitations of this approach are the low penetrance of the beta particles into the tumour and the impossibility of treating multifocal tumours. Systemic administration of radiolabelled MoAbs is not generally applicable, since the quantity of radioactivity that localizes in the solid tumour after intravenous administration is significantly lower than that needed to obtain therapeutic effects (Larson, 1990).

One major limitation of the technique is the frequently unfavourable tumour-to-non tumour distribution of radiolabel, due to the pharmacokinetics of the MoAb, and the binding to non-specific tissues (Goodwin, 1987; Goodwin *et al*, 1988). Among the strategies proposed to overcome these problems, a three-step method (pretargeting) that temporally separates the principal targeting reagent from the radiolabel has given encouraging results (Paganelli *et al*, 1988; 1991; Magnani *et al*, 1996). By exploiting the high affinity of biotin–avidin binding (Wilchek and Bayer, 1984) pretargeting of tumours with biotinylated MoAb and streptavidin has the advantage of decreasing the load of targeting MoAb, while at the same time increasing the specific delivery of radiolabel to the disease site (Hnatowich *et al*, 1987; Sung and van Osdol, 1995). In the first step, the patient is injected with a biotinylated MoAb that binds to an antigen on the tumour cell surface. In pharmacokinetic studies, much of the antibody that does not bind to tumour clears from circulation within 24–48 h (Cremonesi *et al*, 1999) and the optimum tumour to background ratio is achieved. In the second step we inject streptavidin which binds to MoAb on the tumour surface and promote the clearance of the unbound MoAb. This is followed, one to several hours later, by biotinylated effector molecules, often radionuclides. In a phase I–II therapeutic study (Paganelli *et al*, 1999) 48 patients with grade III/IV glioma were treated with anti-tenascin Ab and yttrium-90 labelled biotin. Two months after treatment, 12 out of 48 patients showed objective reduction in tumour mass (three complete remissions) and another 52% of the patients had stable disease. Even 12 months after treatment, 17% of the patients still showed tumour reduction.

These results prompted the present study in which the same approach was used in the adjuvant setting soon after surgery and radiotherapy. The end-points were: (i) time to relapse; and (ii) overall survival.

## METHODS

### Patients

From December 1994 to August 1997 we enrolled 37 high grade glioma patients, 17 with grade III glioma (anaplastic astrocytoma AA; oligodendroglioma OD; oligoastrocytoma OA) and 20 with GBM; 16 were females and 21 were males, age range was 19–83 years. Inclusion criteria were: (a) tenascin expression in the tumour demonstrated immunohistochemically; (b) prior computed tomography (CT) or magnetic resonance (MR) studies revealing no evidence of disease; (c) baseline blood and kidney function tests in normal range; and (d) Karnofsky performance status (KPS) >70%. Exclusion criteria were: (a) KPS <70; (b) presence of macroscopic disease on CT or MR; (c) the presence of a known second cancer; (d) history of allergic or anaphylactic reactions; and (e) pregnancy. The study was approved by the Ethical Committee of the European Institute of Oncology and all patients were informed of the nature, aim and potential risks of the study; treated patients signed a consent form before starting therapy.

The study was designed as an open, controlled, non-randomized one. All patients received surgery and radiotherapy; a central pathology review was performed. Nine were also given platinum-based chemotherapy, before and during radiotherapy, four in the treatment group and five in the control group. Nineteen patients were given the adjuvant RIT with ^90^Y-biotin and thus formed the treatment group. The remaining 18 patients served as controls and received no further treatment. Patients were assigned to the control group for: (i) low availability of monoclonal antibodies, (ii) insufficient expression of tenascin by the tumour, or (iii) a history of allergic reactions; otherwise they would have been assigned to the treatment group and given RIT. Moreover three control patients specifically refused RIT, due to other medical advice (they preferred chemotherapy).

RIT was administered to treatment group patients within a month of baseline assessment by MR or CT scan after 1 month from the end of radiotherapy.

Routine blood tests and liver and renal function tests were performed before and after RIT. Antibodies against murine antibodies, avidin and streptavidin were assayed in serum 30–40 days after RIT. Check-ups, including clinical examination (evaluation of performance status, quality of life, number of seizures, drug intake) and CT or MR scans, were scheduled at 3-month intervals starting 2 months after the end of RIT.

Control patients were also assigned clinical and radiological checks every 3 months. However they complied poorly, so it was not possible to calculate the disease-free interval (DFI) in this group, and only data on survival are available. Detailed characteristics of both groups are reported in Tables 1

**Table 1:**
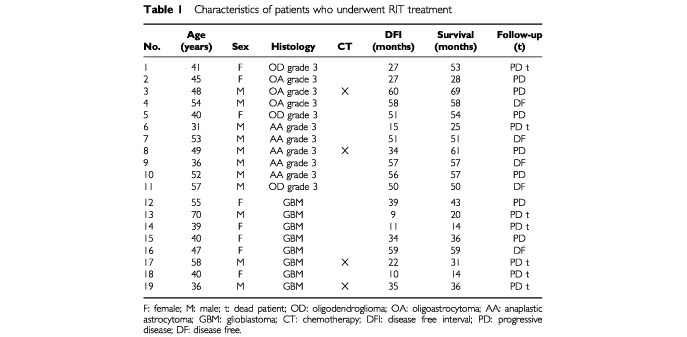
Characteristics of patients who underwent RIT treatment

and 2

**Table 2:**
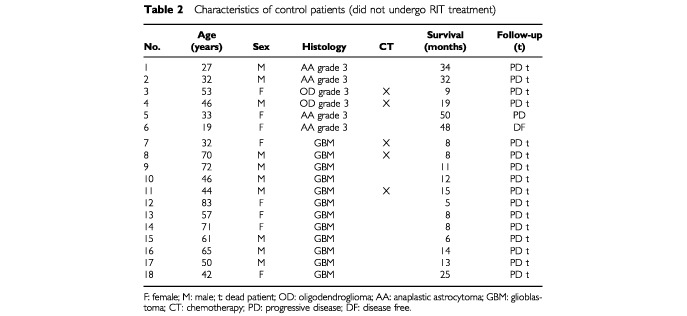
Characteristics of control patients (did not undergo RIT treatment)

.

### Reagents

Anti-tenascin MoAb BC4 was from Sorin Biomedica (Saluggia, Italy). MoAbs were biotinylated by BIO-SPA (Milan, Italy) as described (Paganelli *et al*, 1991). The macrocyclic chelating agent 1,4,7,10-tetraazacyclododecane-N,N′,N′′,N′′′-tetraacetic acid (DOTA), linked to biotin via an amino acid side chain modified by amide N-methylation, was provided by the NeoRx Corporation (Seattle, WA, USA). Yttrium-90 chloride in hydrochloric acid (0.04 M) was purchased from MAP Medical Technologies (Finland).

### Radiolabelling and administration protocols

The radiolabelling procedure and pre-targeting protocol were performed as described previously (Paganelli *et al*, 1999). Briefly, each patient was given 35 mg m^−2^ i.v. of biotinylated MoAb against tenascin. This was followed 24–36 h later by 30 mg of avidin as rapid i.v. bolus (chase), followed 30 min later by 50 mg of streptavidin in 100 ml of saline with 2% human albumin. Sixteen to 18 h later 2 mg of DOTA-biotin ligand, labelled with ^90^Y-chloride (2.2 GBq m^−2^) were injected.

### Data analysis and statistical evaluation

The first post-treatment evaluation by CT or MR was performed 40–60 days later and repeated every 3–4 months. The disease-free interval from diagnosis (that we considered to be surgery) until the first sign of progression on neuroimaging was calculated. Patients with no evidence of progression were considered as censored observations and the DFI was calculated from the date of diagnosis to the date of latest follow-up. For both treated and control patients the time from diagnosis till death was recorded. Patients who were still alive were treated as censored with the date of latest follow-up used to give the survival time.

The two groups of patients, (17 Grade III and 20 Grade IV), were analyzed and are shown separately since they have different prognoses. Kaplan-Meier estimation of survival and a log-rank test were applied to assess any differences between treated and untreated patients. A Cox proportional hazard model was fitted to take into account a possible confounding effect of age (Therneau and Grambsch, 2000). A permutation test was also done as there is a small number of patients in the groups (Davison and Hinkley, 1997). A 5% level of significance was used with 500 Monte Carlo simulations. All the analyses were done in S-PLUS 2000 Professional Release 2 (MathSoft, Inc. 1988–1999 Seattle, USA).

## RESULTS

### Toxicity

In all patients the treatment was well-tolerated. None developed renal or liver alterations during follow-up. One patient, who had previously received monoclonal antibodies and avidin for diagnostic purposes, developed a mild allergic reaction during avidin administration. No patient developed side effects to biotinylated MoAbs or ^90^Y biotin injection.

The presence of antibodies against the injected reagents was assayed before and after treatment. Almost all patients (90%) developed anti-streptavidin antibodies, 70% developed anti-avidin antibodies and 20% developed antibodies against the murine MoAbs. These results are closely similar to those we published previously; in particular they confirm the low frequency of reaction to murine MoAbs and the high immunogenicity of streptavidin (Paganelli *et al*, 1997).

Fifty-eight per cent of patients did not develop haematological toxicity; in the remainder (42%) hematological toxicity was grade I or II. The nadir occurred 3–4 weeks after treatment with full recovery in all cases in 4–6 weeks. There was no correlation between toxicity grade and previous exposure to chemotherapy. These data confirm the safety of administering up to 2.22 GBq m^−2^ of ^90^Y-biotin using our protocol (Paganelli *et al*, 1999).

### Therapeutic effect

The two groups of patients are shown separately since they have different prognoses.

#### Grade IV glioma patients (GBM)

**Figure 1:**
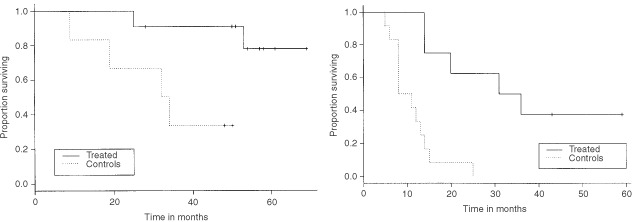
Survival curves comparing treated and control patients. Left: grade III glioma; right grade IV glioma.

Five of the eight grade IV treated patients and all of the 12 controls died. The estimates of median survival in the two groups were, respectively, 33.5 months (95% CI=(20, NA), range=(14–59)) and 8 months (95% CI=(8, NA); range=(5–25)). This difference was significant, (log-rank test=12.6 on 1 d.f.), and the corresponding curves are shown in Figure 1

. The *P*-value from the permutation test was *P*=0.014. The estimated median Disease Free Interval, in the eight treated patients, was 28 months (95% CI=(11, NA), range=(9–59)).

Figure 2

**Figure 2:**
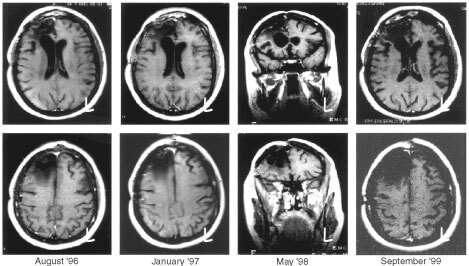
MR study of a female patient (47 years old; No. 16) who had surgery for glioblastoma in April 1996: set of images starting from the baseline evaluation to the last control (September 1999): no evidence of relapse. The patient is scheduled for a new MR in summer 2001.

is an example of long lasting response to adjuvant RIT in a patient who had surgery for glioblastoma, showing a good response to RIT.

#### Grade III glioma patients

Only two of the 11 grade III treated patients died, so the estimate of the median overall survival is not available. Among the controls, such an estimate was 33 months (95% CI=(19, NA), range=(9–50)). The difference in survival was significant (log-rank test=6.1 on 1 d.f.) and the K-M estimated curves are shown in Figure 1. The *P*-value from the permutation test was *P*=0.002. The estimated median Disease Free Interval, in the 11 treated patients, was 56 months (95% CI=(34, NA), range=(15–60)).

## DISCUSSION

Despite their local pattern of growth and progression, malignant gliomas are the third most important cause of cancer death in the 15–34 age range (Walker *et al*, 1985; Mahaley *et al*, 1989; Salcman, 1990) with median survival times of 6–12 months for GBM and 15–27 months for grade III glioma (Florell *et al*, 1992).

Current recommended treatment for high grade gliomas is surgical resection followed by radiation therapy (RT) (Leibel and Sheline, 1987). However even with complete macroscopic debulking and external RT at doses above 60 Gy (Salazar *et al*, 1979), the tumour inevitably recurs, probably due to the presence of malignant cells at the edge of surgical cavity which are not killed by irradiation (Burger, 1983). Despite the use of aggressive combined therapies, the poor prognosis for these patients reflects lack of sufficiently potent agents with adequate tumour specificity.

In the present study, our main objectives were the evaluation of the disease free interval and the overall survival, following RIT in combination with surgery, radiotherapy and chemotherapy. The fact that our patient population was a selected one (either active or control; the macroscopically disease free status; good clinical conditions) enhances the overall clarity of the present study because it simplifies the interpretation of the data and facilitates the comparison with trials involving different therapeutical approaches. The absence of macroscopic disease after surgery, a good KPS are, of course, favourable prognostic factors; although they do not exclude from recurrence and patients remain with a short life expectancy in both groups.

The three-step method we have developed, which exploits the avid and specific binding of the avidin/biotin system, targets radionuclides to specific MoAbs already previously localized on the tumour cells (Wilchek and Bayer, 1984). Pre-clinical (Hnatowich *et al*, 1987; Paganelli *et al*, 1990) and clinical (Kalofonos *et al*, 1990; Paganelli *et al*, 1994) studies have shown that multi-step avidin/biotin methods result in improved localization of radionuclides to tumours compared to radiolabelled MoAbs (Sung and van Osdol, 1995; Beaumier *et al*, 1995). Furthermore encouraging results were obtained in a phase I-II clinical study (Paganelli *et al*, 1999).

The results of this pilot study are promising, even though they relate to a small patient population. Four of 11 grade III glioma patients were disease free at latest follow-up and eight patients have been followed for more than 30 months without progression. More remarkable were the outcomes in the treated GBM patients, in whom DFI and consequently life expectancy was much improved relative to our controls. The group has a DFI of 28 months and overall survival of 33.5 months from diagnosis with one of the original eight patients disease free and in follow-up at latest follow-up. Survival times in both the treated grade III (log rank test=6.1, *P*=0.013) and treated GBM patients (log rank test=12.6, *P*<0.001) were significantly longer than in the corresponding controls. The fitted models accounted for a potential effect of age and no difference was found, overall, in the two groups. Although the controls for Grade IV were slightly older and the controls for Grade III were slightly younger than their respective treated patients, such a difference was not statistically significant. A treatment effect is, in fact, still evident after adjusting for age.

Clearly if these results were from a randomized trial or from a greater group, they would constitute highly convincing evidence that our 3-step RIT protocol is effective in improving survival and DFI in these almost invariably fatal malignancies. Therefore our results are only suggestive of an improvement in survival. Nevertheless we believe that this non-randomized study provides useful information, particularly in the context of a very poor prognosis disease such as GBM, in which non-randomized studies are of use, as GBM is a rare advanced and severe cancer (Altman and Bland, 1999). In particular the results in our glioblastoma patients strongly suggest RIT should be more widely used as adjuvant therapy in this condition.

The low toxicity of high dose ^90^Y-biotin with three-step pre-targeting strategy is an important advantage over the use of directly labelled antibodies (Paganelli *et al*, 1988). Using this approach the radiolabelled material is cleared quickly from the blood-pool, has favourable biodistribution and is rapidly excreted via the kidneys.

This entails acceptable absorbed doses to normal organs (kidneys: 1.6±1.1; liver: 0.3±0.2 mGy MBq^−1^) including red marrow (0.10±0.05 mGy MBq^−1^) (Cremonesi *et al*, 1999). Furthermore, one of the main therapeutic advantages of radionuclides, compared to other cytotoxic agents, is their potential for overcoming the problem of tumour heterogeneity: high grade glioma contains not only viable areas but also hypoxic and necrotic cells. Therefore a radionuclide with high energy emission, such as ^90^Y (Eβ_MAX_= 2.2 MeV) that penetrate up to 11 millimetres of tissue can kill tumour cells that have no MoAbs on their surface (crossfire effect).

A number of points arise from the results of this trial. Firstly, RIT is confirmed as highly active against malignant glioma, yet does not cause major adverse events, as previously described (Paganelli *et al*, 1999). Secondly the effect RIT on GBM is interesting: it considerably prolonged DFI and overall survival relative to the untreated group. We also believe that there is room to further improve the efficacy of the method by modifying its timing. It may be advantageous to administer RIT immediately after debulking surgery, in order to exploit the greater permeability of the blood–brain barrier at that time. This would be expected to expose more malignant cells to the radionuclide and hence limit the local spread of the cancer.

We emphasize however that RIT should be used as part of a combined modality approach. To illustrate this we note the results of a combined treatment in a patient with advanced oropharyngeal carcinoma treated with surgery, radio-chemotherapy and three-step radioimmunotherapy (Paganelli *et al*, 1998). In this patient a partial tumour response was achieved after surgery and radio-chemotherapy. After administration of RIT the patient presented a complete response that lasted more than 17 months.

The combined modality approach to treating brain tumours was introduced about 30 years ago; it remains the most effective approach we have. The promise is that the combination of surgery, radiotherapy, chemotherapy and RIT may provide, at last, a way of increasing life expectancy in high grade glioma patients (O'Reilly *et al*, 1993).

In conclusion, three-step RIT could have an important role as adjuvant treatment in high grade gliomas, as it interferes with progression, prolonging time to relapse and overall survival. Presumably, based on these results, this technique should be performed in an adjuvant setting in a larger number of patients with high grade glioma and a randomized trial is fully justified.

## References

[bib1] AltmanDGBlandJM1999 Statistics notes. Treatment allocation in controlled trials: why randomise? BMJ 31871921209 10221955 10.1136/bmj.318.7192.1209 PMC1115595

[bib2] BeaumierPLAxworthyDBFritzbergARHylaridesMDMalletRWTheodoreLJGustavsonLMSuF-MRenoJM1995 The pharmacology of pretargeting components: optimizing therapeutic targeting Q J Nucl Med 39 20

[bib3] BurgerPC1983 Pathologic anatomy and CT correlations in the glioblastoma multiforme Appl Neurophysiol 46 180187 6322685

[bib4] CremonesiMFerrariMChinolMStabinMGGranaCPriscoGRobertsonCTosiGPaganelliG1999 Three-step radioimmunotherapy with yttrium-90-biotin: dosimetry and pharmacokinetics in cancer patients Eur J Nucl Med 26 110120 9933344 10.1007/s002590050366

[bib5] CulverKWRamZWallbridgeSIshiiHOldfieldEHBlaeseRM1992 In vivo gene transfer with retroviral vector-producer cells for treatment of experimental brain tumors Science 256 15501552 1317968 10.1126/science.1317968

[bib6] DavisonACHinkleyDV1997 Bootstrap methods and their application(Chapter 4)Cambridge Series in Statistical and Probabilistic Mathemathics.Cambridge, UK: Cambridge University Press

[bib7] FlorellRCMacdonaldDRIrishWDBernsteinMLeibelSAGutinPHCairncrossJG1992 Selection bias, survival, and brachytherapy for glioma J Neurosurg 76 179183 1730945 10.3171/jns.1992.76.2.0179

[bib8] GoodwinDA1987 Pharmacokinetics and antibodies J Nucl Med 28 13581362 3612296

[bib9] GoodwinDAMearesCFMcCallMJMcTigueMChaovapongW1988 Pre-targeted immunoscintigraphy of murine tumors with indium-111-labeled bifunctional haptens J Nucl Med 29 226234 3346734

[bib10] HnatowichDJVirziFRusckowskiM1987 Investigations of avidin and biotin for imaging applications J Nucl Med 28 12941302 3612292

[bib11] KalofonosHPRusckowskiMSiebeckerDASivolapenkoGBSnookeDLavenderJPEpenetosAAHnatowichDJ1990 Imaging of tumour in patients with indium-111-labeled biotin and streptavidin-conjugated antibodies: preliminary communication J Nucl Med 31 17911796 2230992

[bib12] LarsonSM1990 Clinical radioimmunodetection, 19781988: overview and suggestions for standardization of clinical trialsCancer Res 50 892898 2404583

[bib13] LeibelSAShelineGE1987 Radiation therapy for neoplasms of the brain J Neurosurg 66 122 3023563 10.3171/jns.1987.66.1.0001

[bib14] MagnaniPPaganelliGModoratiGZitoFSonginiCSudatiFKochPMaeckeHRBrancatoRSiccardiAGFazioF1996 Quantitative comparison of direct antibody labeling and tumor pretargeting in uveal melanoma J Nucl Med 37 967971 8683323

[bib15] MahaleyJrMSMettlinCNatarajanNLawsJrERPeaceBB1989 National survey of patterns of care for brain-tumor patients J Neurosurg 71 826836 2585073 10.3171/jns.1989.71.6.0826

[bib16] O'ReillySMNewslandESGlaserMGBramptonMRice-EdwardsJMIllingworthRDRichardsPGKennardCColquhounIRLewisP1993 Temozolomide: a new oral cytotoxic chemotherapeutic agent with promising activity against primary brain tumours Eur J Cancer 29 940942 10.1016/s0959-8049(05)80198-4 8499146

[bib17] PaganelliGChinolMMaggioloMSidoliACortiABaroniSSiccardiAG1997 The three-step pretargeting approach reduces the human anti-mouse antibody response in patients submitted to radioimmunoscintigraphy and radioimmunotherapy Eur J Nucl Med 24 350351 9143478 10.1007/BF01728778

[bib18] PaganelliGGranaCChinolMCremonesiMDe CiccoCDe BraudFRobertsonCZurridaSCasadioCZoboliSSiccardiAGVeronesiU1999 Antibody-guided three-step therapy for high grade glioma with yttrium-90 biotin Eur J Nucl Med 26 348357 10199940 10.1007/s002590050397

[bib19] PaganelliGMagnaniPZitoFLucignaniGSudatiFTurciGMottiETerreniMPolloBGiovannelliMCanalNScottiGComiGKochPMaeckeHRFazioF1994 Pre-targeted immunodetection in glioma patients: tumour localization and single-photon emission tomography imaging of [^99m^Tc]PnAO- biotin Eur J Nucl Med 21 314321 8005155 10.1007/BF00947966

[bib20] PaganelliGMagnaniPZitoFVillaEStellaMSudatiFLopalcoLRossettiCMalcovatiMChiolerioFSeccamaniESiccardiAGFazioF1991 Three-step monoclonal antibody tumour targeting in carcinoembryonic antigen-positive patients Cancer Res 51 59605966 1933860

[bib21] PaganelliGOrecchiaRJereczek-FossaBGranaCCremonesiMde BraudFTradatiNChinolM1998 Combined treatment of advanced oropharyngeal cancer with external radiotherapy and three-step radioimmunotherapy Eur J Nucl Med 25 13361339 9724386 10.1007/s002590050305

[bib22] PaganelliGPervezSSiccardiAGRowlinsonGDeleideGChiolerioFMalcovatiMScassellatiGAEpenetosAA1990 Intraperitoneal radio-localization of tumors pre-targeted by biotinylated monoclonal antibodies Int J Cancer 45 11841189 2351490 10.1002/ijc.2910450632

[bib23] PaganelliGRivaPDeleideGClivioAChiolerioFScassellatiGAMalcovatiMSiccardiAG1988 In vivo labelling of biotinylated monoclonal antibodies by radioactive avidin: a strategy to increase tumour radiolocalisation Int J Cancer 2 121125 10.1002/ijc.2910410727 3162440

[bib24] RivaPAristaATisonVSturialeCFranceschiGSpinelliARivaNCasiNMoscatelliGFrattarelliM1994 Intralesional radioimmunotherapy of malignant gliomas: an effective treatment in recurrent tumors Cancer 73 10761082 8306250 10.1002/1097-0142(19940201)73:3+<1076::aid-cncr2820731347>3.0.co;2-z

[bib25] RothJACristianoRJ1997 Gene therapy for cancer: what have we done and where are we going? J Natl Cancer Inst 88 2139 10.1093/jnci/89.1.21 8978404

[bib26] SalazarORubinPFeldsteinMPizzutielloR1979 High dose radiation therapy in the treatment of malignant gliomas: final report Int J Rad Oncol Biol Phys 5 17331740 10.1016/0360-3016(79)90554-6 231023

[bib27] SalcmanM1990 Epidemiology and factors affecting survivalInMalignant Cerebral Glioma,Apuzzo MLJ (ed),pp95110American Association of Neurological Surgeons.Illinois: Park Ridge

[bib28] SungCvan OsdolWW1995 Pharmacokinetic comparison of direct antibody targeting with pretargeting protocols based on streptavidin-biotin binding J Nucl Med 36 867876 7738665

[bib29] TherneauTMGrambschPM2000 Modeling Survival Data.pp.776Heidelberg, Germany: Springer

[bib30] WalkerAERobinsMWeinfeldFD1985 Epidemiology of brain tumors: the national survey of intracranial neoplasms Neurology 35 219226 3969210 10.1212/wnl.35.2.219

[bib31] WilchekMBayerEA1984 The avidin-biotin complex in immunology Immunol Today 5 3943 25291372 10.1016/0167-5699(84)90027-6

[bib32] ZalutskyMRMoseleyRPCoakhamHBColemanREBignerDD1989 Pharmacokinetics and tumour localization of ^131^I-labeled anti-tenascin monoclonal antibody 81C6 in patients with gliomas and other intracrianal malignancies Cancer Res 49 28072813 2469537

